# The Protective Effect of Anethole against Renal Ischemia/Reperfusion: The Role of the TLR2,4/MYD88/NFκB Pathway

**DOI:** 10.3390/antiox11030535

**Published:** 2022-03-11

**Authors:** Maged Elsayed Mohamed, Mahmoud Kandeel, Hany M. Abd El-Lateef, Hossam S. El-Beltagi, Nancy S. Younis

**Affiliations:** 1 Department of Pharmaceutical Sciences, College of Clinical Pharmacy, King Faisal University, Al-Ahsa 31982, Saudi Arabia; nyounis@kfu.edu.sa; 2 Department of Pharmacognosy, College of Pharmacy, Zagazig University, Zagazig 44519, Egypt; 3 Department of Biomedical Sciences, College of Veterinary Medicine, King Faisal University, Al-Ahsa 31982, Saudi Arabia; mkandeel@kfu.edu.sa; 4 Department of Pharmacology, Faculty of Veterinary Medicine, Kafrelsheikh University, Kafrelsheikh 33516, Egypt; 5 Department of Chemistry, College of Science, King Faisal University, Al-Ahsa 31982, Saudi Arabia; hmahmed@kfu.edu.sa; 6 Department of Chemistry, Faculty of Science, Sohag University, Sohag 82524, Egypt; 7 Agricultural Biotechnology Department, College of Agriculture and Food Sciences, King Faisal University, Al-Ahsa 31982, Saudi Arabia; helbeltagi@kfu.edu.sa; 8 Biochemistry Department, Faculty of Agriculture, Cairo University, Giza 12613, Egypt

**Keywords:** anethole, anti-apoptotic, anti-inflammatory, anti-oxidant, renal ischemia/reperfusion, in silico

## Abstract

Background: Anethole is the principle essential oil component of anise and fennel. Renal ischemia/reperfusion (RIR) is one of the utmost imperative reasons for acute kidney injury and often associated with high mortality rate. The aim of this study is to investigate the protective effect of anethole on RIR status, exploring the involved mechanisms. Methods: RIR was accomplished by bilateral renal pedicle clamping for 45 min, after which the clamps were removed to achieve the reperfusion phase. Rats were randomized into five groups; Sham, Sham + anethole, RIR, and finally RIR + anethole (125 mg/kg or 250 mg/kg) groups. Animals were given anethole (in specified groups in doses) for 14 days before RIR. Results: RIR-experienced animals developed renal injury evidenced by diminished renal function and histopathological alteration. RIR induced severe oxidative, inflammatory, and apoptotic status within renal tissue. Pre-RIR management with anethole enhanced renal morphology and improved renal function. Anethole amplified GSH content and SOD, CAT, and GPx activities and lowered MDA. Anethole reduced gene and protein expression levels of HMGB1, TLR2, TLR4, MYD88, and NFκB. Anethole distinctly dropped TNF-α, IFN-γ, and MCP-1 levels, increased IL-10, and diminished caspase 3 and 9, reflecting its anti-inflammatory and anti-apoptotic actions. Conclusion: Anethole displayed anti-inflammatory, anti-oxidant, and anti-apoptotic actions against RIR-induced injury. Anethole exhibited renal protective actions, which could be through inhibiting the HMGB1/TLR2, 4/MYD88/NFκB pathway. These results could suggest anethole as a protective agent against RIR.

## 1. Introduction

During certain clinical settings such as renal transplant, hemi-nephrectomy, and renal artery occlusion, renal blood supply is temporarily declined or even impaired, causing renal ischemia/reperfusion (RIR) injury [1]. RIR is one of the highest imperative reasons for acute kidney injury and mortality [2]. RIR injury involves oxidative stress [3], inflammation [4], and apoptosis [5]. Molecular pathways involved in RIR are diverse, including vascular permeability enhancement, inflammatory responses and cytokine release instigation, penetration of leukocytes to the damaged tissue, ATP exhaustion, and extracellular acidosis [6]. RIR injury arises because of two sequential events. Initially, during ischemia, a hypoxia period occurs, resulting in free oxygen radical production. Oxygen-derived free radical intensification outweighs renal detoxification capability, leading to lipid peroxidation as well as cellular membrane destruction [3]. This process prompts pro-inflammatory reactions, leading to renal cells apoptosis. Subsequently, damaged cells release intracellular damage associated molecular patterns (DAMPs), for instance, the high-mobility group (HMG), which are distinguished by innate receptors to induce further inflammation [7]. High-mobility group box 1 (HMBG1) is a member of the HMG protein family that activates Toll-like receptors (TLRs), specifically TLR2 and TLR4, to trigger pro-inflammatory cytokine expression [8,9]. TLRs, specifically TLR2, TLR4, and TLR9, are triggered via DAMPs that can accumulate during ischemia. Together the innate immune system and TLRs prompt the development of ischemia/reperfusion injury in diverse laboratory ischemic conditions, including heart [10], liver [4], brain [11], and kidney [12]. Oxidative stress, inflammation, and apoptosis are all tangled together, leading to a dreadful deterioration circle. Compounds that possess antioxidant, anti-inflammatory, and anti-apoptotic effects have been demonstrated to alleviate RIR injury to a certain degree [1,2,5,13].

**Figure 1:**
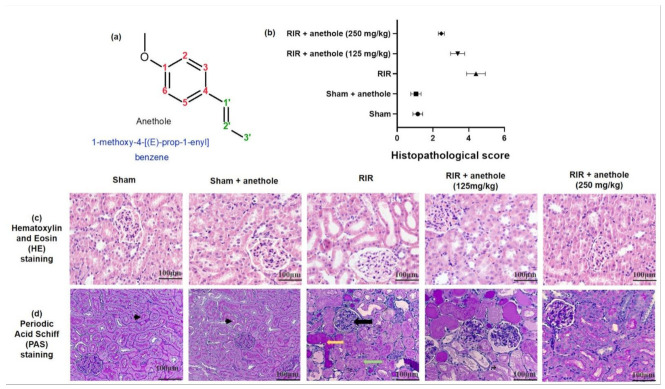
(**a**) Anethole chemical structure, (**b**) quantitative assessment of renal damage score, (**c**,**d**) histopathological analysis displaying the protective action of anethole (125 and 250 mg/kg) on the renal tissues challenged through RIR stained with hematoxylin and eosin (H&E) and periodic acid Schiff (PAS) stain, respectively. The black arrows show glomeruli collapsed with focal necrosis. The yellow arrow shows hyaline accumulation. The green arrow illustrates the necrotic tubule.

Anethole (1-methoxy-4-[(E)-prop-1-enyl] benzene; Figure 1a) is a phenylpropanoid, typically attained from anise (*Pimpinella anisum*), sweet anise (*Foeniculum vulgare*), and star anise (*Illicium verum*) essential oils [14]. Anethole administration demonstrated no toxic effects or injurious on the liver [15]. Anethole was confirmed to possess anti-metastatic [16], antioxidant, anti-inflammatory and gastroprotective [17], antifungal [18], antiviral [19], antihypernociceptive [15], local anesthetic [20], vasodilation, and antihypertensive [21] activities. Moreover, anethole exhibited anti-inflammatory properties in the non-immune acute inflammation animal models [15], protection against hepatic ischemia/reperfusion injury in mice [22], oxygen–glucose deprivation/re-oxygenation in cortical neuronal cells [23], and cyclophosphamide-induced immunity suppression in mice [14]. Additionally, anethole exerted antimetastatic activity via inhibition of the matrix metalloproteinase 2/9 and AKT/mitogen-activated kinase/NFκB pathways [16]. Anethole showed anti-inflammatory effects in different inflammation models induced by carrageenan [15] and complete Freund’s adjuvant and in chronic inflammation models [24]. 

The intention of the existing study was to assess the outcome of anethole, as a phenylpropanoid natural compound, on renal biochemical as well as histological features following RIR. Moreover, we tried to estimate the underlying cellular and molecular pathways involved in this shielding effect of anethole. 

## 2. Materials and Methods

### 2.1. Animals

The Experimental Animal Research Centre, King Saud University, Riyadh, KSA, was the source from which Wistar male rats (age: 4–5 weeks; weight: 220–250 g) were acquired. All the animals were maintained with typical laboratory food and water ad libitum in a ventilated cage system (12 h light/dark cycle, 20.3–23.1 °C) throughout the whole experiment. 

### 2.2. Ethical Approval Declaration

The Institutional Animal Care and Use Committee of the King Faisal University allowed and permitted the experimental protocol (KFU-REC-2022-JAN-EA000361). All the animal handling and experiments as well as tests were executed according to the appropriate guidelines and regulations of the Ethical Conduct for Use of Animals in Research at King Faisal University.

### 2.3. Induction Renal Ischemia/Reperfusion (RIR) Injury

Fasted rats (for 8 h) were anesthetized using ketamine (75 mg/kg) and xylazine (8 mg/kg) intraperitoneally. During the whole surgeries, animals’ body temperatures were maintained at 36–38 °C via retaining the animals on a surgical heating pad. Under sterilized surroundings, a midline opening was executed to detect the kidneys. Both left and right pedicles were occulted bilaterally with two non-traumatic vascular clamps for 45 min, and then the kidney color turned pale indicating establishment of renal ischemia [5,25]. After 45 min, the clamps was detached, and the kidneys were observed for five min till the color turned reddish brown, which indicated the occurrence of the reperfusion phase. The abdominal wall and skin were sealed via sutures. Twenty-four hours post-surgery, the rats were anesthetized again with the same doses mentioned above. Blood samples were acquired from the hearts, centrifuging (20 min, 5000× *g*) to attain serum, which were stored at −20 °C for subsequent biological determinations. The right kidney was rapidly detached and homogenized and kept at −80 °C for biochemical investigations, and the left one was preserved in 10% formalin for histopathological and immunohistochemical examination.

### 2.4. Experimental Design

Rats were randomized into five groups (*n =* 6) following the experimental design stated in Table 1. The sham group of animals was only subjected to bilateral renal artery separation but without artery clamping. The sham + anethole group was treated similarly to the sham group; however, the animals were given anethole (trans-anethole: Catalogue No; 117870, Merck (Sigma-Aldrich, St. Louis, MO, USA)), in a dose of 250 mg/kg, dissolved in 1% carboxymethylcellulose (CMC) in saline orally via gastric lavage for 14 days before the surgery. The RIR group represented the animal group where the renal injury was performed in rats by renal ischemia/reperfusion surgery (RIR). In RIR + anethole groups, the animals were administered anethole 125 mg/kg or 250 mg/kg orally for 14 day before the renal ischemia/reperfusion surgery, as mentioned above, and subjected to the RIR surgery [26,27].

### 2.5. Evaluations Using Histopathological and Immunohistochemical Investigations

Kidney samples were fixed, and paraffin bees wax tissue blocks were cut to obtain 4 μm in thickness sections. The paraffin embedded tissue sections were placed on glass slides, deparaffinized, and stained via hematoxylin and eosin (H&E) and periodic acid Schiff (PAS) stain to be examined under a light microscope. Two blinded pathologists assessed the histological renal injury. Renal sections were scored using a scale intended to estimate the grade of renal tubulointerstitial damages. The scoring system used was 0 (no damage), 1 (less than 10%), 2 (11–25%), 3 (26–45%), 4 (46–75%), and 5 (more than 76%), as mentioned earlier [28]. A minimum of 10 fields (200× magnification) for each kidney section was inspected and assigned for severity of changes.

For the immunohistochemistry (IHC) procedure, the expression of HMGB1 and NFκB were investigated as mentioned earlier [29], using HMGB1 and NFκB antibodies (Thermo Fisher Scientific, Cambridge, UK), and goat anti-rabbit-horseradish peroxidase (HRP) conjugated IgG antibody (Cat. no. ab6721; Abcam, Eugene, OR, USA). A digital camera (Nikon Instruments Inc., Melville, NY, USA) was operated to visualize the renal sections. Positive cells were counted under 400× magnification, observing ten consecutive non-overlapping fields per animal in a blinded manner. 

### 2.6. Evaluation of Renal Function

Renal function was evaluated via assessing serum levels of creatinine (Cr; ab700460), uric acid (ab65344), blood urea nitrogen (BUN; ab83362), lactate dehydrogenase (LDH; ab102526), and kidney injury molecule-1 (Kim-1; ab119597) using the specified colorimetric or ELISA kit obtained from Abcam Inc. (Waltham, MA, USA), agreeing with the manufacturer’s practices consuming a spectrophotometer (LEICA UNISTAT^®^; Leica Inc., Allendale, NJ, USA). 

### 2.7. Evaluation of Renal Oxidative Stress Status 

Malondialdehyde (MDA; ab238537), glutathione peroxidase (GPx; ab102530), and glutathione content (GSH; ab239727) ELISA kits were procured from Abcam Inc. (Cambridge, UK). Superoxide dismutase (SOD; MBS036924) and catalase (CAT; MBS726781) ELISA kits were acquired from My BioSource (San Diego, CA, USA) and performed in agreement with the manufacturer’s directions. 

### 2.8. Evaluation of Toll-like Receptors (TLR) Pathway Gene Expression 

Gene expression for the TLR pathway, HMGB-1, TLR2, TLR4, myeloid differentiation primary response gene 88 (MYD88), and NFκB were quantified via real-time PCR (qPCR) consuming the primers’ sequences, shown in Table 2, in agreement with the method described elsewhere [30]. Briefly, RNA was isolated and purified using a Trizol reagent kit (Invitrogen, Carlsbad, CA, USA), then a reverse transcription polymerase chain reaction (RT-PCR) kit (TaKaRa, Kusatsu, Shiga, Japan) to reverse transcription reaction following the manufacturer’s procedures. qPCR was applied using a SYBR ExScript RT-PCR kit, and quantification examinations were accomplished via an Opticon-2 Real-time PCR reactor (MJ Research, Capital Court, Reno, NV, USA). qPCR results were obtained using Step PE Applied Biosystems (Waltham, MA, USA) software. Relative gene expression data were calculated as mentioned earlier by the Livak et al. (2001) [31] method (2^−ΔΔCq2^) and presented as a fold change. Target gene expressions were assessed and related to the reference gene (β-actin), and the results are shown in the figures as relative expression. 

### 2.9. Evaluation of Toll-like Receptors (TLR) Pathway Protein Expression 

Western blot was performed according to the method described previously by Younis et al. (2019) [30] using the same antibodies and techniques specified in the reference. 

### 2.10. In Silico Evaluation of Toll-like Receptors (TLR) Interaction with Anethole 

The TLR4 co-receptor mechanism and the key interactions that govern the molecular recognition of ligands were investigated by molecular docking. The PDB ID 4G8A was used to investigate the potential binding pattern of anethole with the TLR4-MD-2 complex. Protein preparation was performed by the Schrodinger suite protein preparation wizard. All missing atoms and loops were corrected, the structure was optimized and energy minimized. The docking grid was built using the bound LPS ligand as a guide. Anethole 2D structure was obtained from PubChem (https://pubchem.ncbi.nlm.nih.gov/, accessed on 11 November 2021) and 3D optimized by LigPrep software (Schrodinger LLC, New York, NY, USA). 

### 2.11. Evaluation of Inflammatory Indicators

Inflammation markers including interferon gamma (IFN-γ; ab46107), monocyte chemoattractant protein-1 (MCP-1; ab219045), tumor necrosis factor alpha (TNF-α; ab239425), and interleukin 10 (IL-10; ab214566) ELISA kits were obtained from Abcam Inc. (Cambridge, UK) and executed as stated in the manufacturers’ instructions. 

### 2.12. Evaluation of Apoptotic Indicators

The actions of anethole administration on the apoptotic markers, such as caspase-3 (ab39401) and caspase 9 (ab65608), were evaluated via kits acquired from Abcam Inc. (Cambridge, UK), following the producers’ directions. 

### 2.13. Statistical Analysis

Data were shown as mean ± SD. Multiple comparisons was performed using one-way ANOVA followed by Tukey–Kramer as a post hoc test. The 0.05 level of probability was used as the significance level. Statistical analyses were executed using GraphPad software (version 8, San Diego, CA, USA).

## 3. Results

### 3.1. Anethole Averts RIR-Induced Renal Histological Alterations

Light microscopic evaluation of sham groups exposed consistent normal morphology of the renal tissue; for instance, black arrowheads point to intact brush borders, while the renal section attained from animals that underwent RIR revealed severe acute tubular damage, as demonstrated in Figure 1c,d. In PSA sections, the black arrow showing glomeruli collapsed with focal necrosis, yellow arrows representing hyaline accumulation, and green arrows displaying the necrotic tubule. These histological features involved tubular cell swelling, congestion, tubular dilatation, moderate to severe necrosis, and hyaline casts. Administration of anethole prior to RIR enhanced renal morphology, as indicated by the histopathological score (Figure 1b), and showed better glomeruli with less necrosis as well as less edema and dilatation of the tubular cells. However, the casts were still present, especially with a lower concentration of the anethole group RIR + anethole (125 mg/kg).

### 3.2. Anethole Averts RIR-Induced Renal Function Alterations

**Figure 2:**
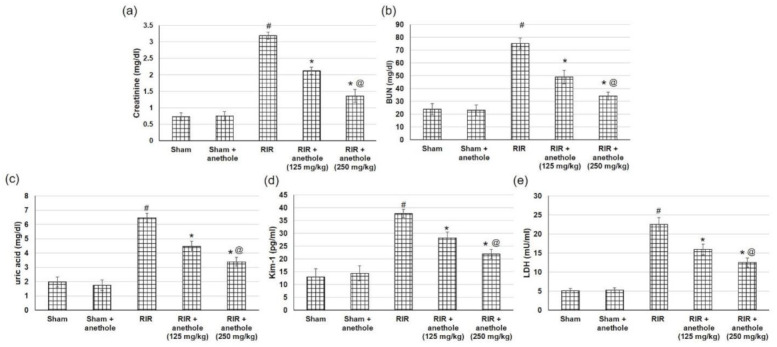
Effects of anethole (125 and 250 mg/kg) administration for 14 days prior to renal ischemia/perfusion (RIR) on the renal function assessment, including (**a**) creatinine, (**b**) BUN, (**c**) uric acid, (**d**) Kim-1, and (**e**) LDH in RIR-induced injury. All values are stated as mean ± SD. # designates statistically significant compared to sham group, * designates statistically significant compared to RIR group, and @ designates statistically significant compared to RIR + anethole 125 mg/kg group (*p* < 0.05) using one-way ANOVA followed by Tukey’s post hoc test.

Renal elimination of BUN and Cr was weakened; thus, they were maintained in the blood due to RIR-induced injury. Likewise, LDH was released into the bloodstream after tissue injury. Furthermore, Kim-1, which is a biomarker for kidney proximal tubule damage [32], was measured. As revealed in Figure 2, serum Cr, BUN, uric acid, LDL, and Kim-1 levels were all significantly increased in the RIR group compared with in the sham group, reaching 3.19 ± 0.1 vs. 0.72 ± 0.12, 75.27 ± 4.3 vs. 23.91 ± 4.2, 6.47 ± 0.35 vs. 1.97 0.36, 22.48 ± 1.8 vs. 5.14 ± 0.54, and 37.77 ± 1.6 vs. 13.04 ± 3.1 mg/dL, respectively. On the other hand, anethole (125 and 250 mg/kg) administration prior to renal ischemia/reperfusion (RIR) surgery significantly lowered the increased levels of Cr, BUN, uric acid, LDL, and Kim-1 related to the RIR group (*p* < 0.05). Additionally, RIR + anethole (250 mg/kg) showed significant lower levels of serum Cr, BUN, uric acid, LDL, and Kim-1 in relation to the RIR + anethole (125 mg/kg) group (*p* < 0.05). These outcomes specify that administration of anethole prior to RIR may efficiently avert RIR-induced renal function alterations.

### 3.3. Anethole Improves RIR Induced Renal Oxidative Stress

**Figure 3:**
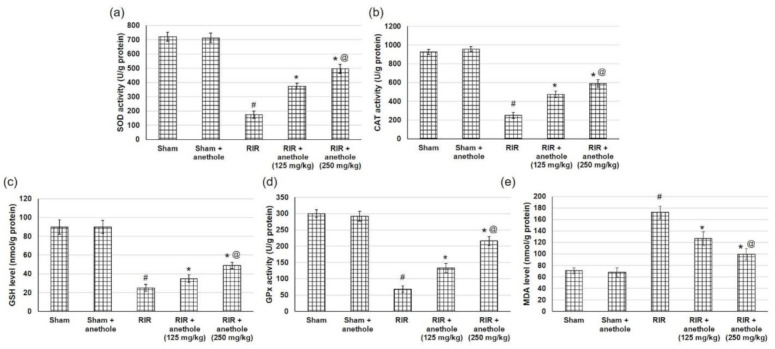
Effects of anethole (125 and 250 mg/kg) administration for 14 days prior to renal ischemia/perfusion (RIR) on the renal oxidative stress (**a**) SOD, (**b**) CAT, (**c**) GSH, (**d**) GPx, and (**e**) MDA in RIR-induced injury. All values are stated as mean ± SD. # designates statistically significant compared to sham group, * designates statistically significant compared to RIR group, and @ designates statistically significant compared to RIR + anethole 125 mg/kg group (*p* < 0.05) using one-way ANOVA followed by Tukey’s post hoc test.

Reoxygenation following ischemia causes tissue oxidative stress during RIR. To appraise the oxidative stress accompanying RIR injury, numerous antioxidant enzymes activities were measured. The current study verified that in RIR rats, the activities of SOD, CAT, and GPx were considerably declined, whereas anethole administration enhanced RIR-induced renal attenuation in SOD, CAT, and GPx activities (Figure 3a,b,d). Additionally, RIR caused an obvious reduction in renal GSH content related to the sham group (24.98 ± 4.03 vs. 89.82 ± 7.8 nmol/g protein), whereas anethole (125 and 250 mg/kg) markedly amplified GSH renal content, reaching 34.71 ± 4.03 and 48.81 ± 3.5 nmol/g protein, respectively (*p* < 0.05), as displayed in Figure 3c. Furthermore, MDA levels were augmented in RIR-experienced animals compared to the sham animals, reaching 172.31 ± 11.6 vs. 70.66 ± 5.07, while anethole administration (125 and 250 mg/kg) prior to RIR obviously prevented the amplified levels of MDA, indicating lower lipid peroxidation, reaching 126.8 ± 12.01 and 99.52 ± 9.5 nmol/g protein, respectively, as presented in Figure 3e. Furthermore, the RIR + anethole (250 mg/kg) group showed significantly higher levels of SOD, CAT, and GPx activities and GSH renal content as well as lowered MDA levels when compared to the RIR + anethole (125 mg/kg) group (*p* < 0.05). All these results together suggest that RIR-induced oxidative stress may be managed by anethole due to its efficient antioxidant ability. 

### 3.4. Anethole Averts RIR Induced Renal Toll like Receptors (TLR) Alterations 

**Figure 4:**
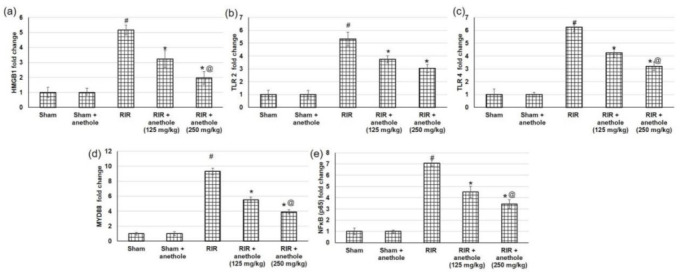
Effects of anethole (125 and 250 mg/kg) administration for 14 days prior to renal ischemia perfusion (RIR) on the renal gene (mRNA) expression of (**a**) HMGB1, (**b**) TLR2, (**c**) TLR4, (**d**) MYD88, and (**e**) NFκB in RIR-induced injury. All values are stated as mean ± SD. # designates statistically significant compared to sham group, * designates statistically significant compared to RIR group, and @ designates statistically significant compared to RIR + anethole 125 mg/kg group (*p* < 0.05) using one-way ANOVA followed by Tukey’s post hoc test.

Injury caused by RIR produces DAMP proteins such as HMGB1, which signal through TLRs, particularly TLR2 and TLR4, to trigger numerous inflammatory mediators including NFκB [33]. Therefore, to examine the underlying mechanism of anethole on RIR, we tried to explore the mRNA and protein expression levels of HMGB1, TLR2, TLR4, and their adaptor protein MYD88 and finally NFκB to have an overview on the whole pathway. HMGB1 mRNA expression level was increased when related to sham-operated rats, whereas anethole pretreatment lowered RIR-induced augmented HMGB1 expression, as seen in Figure 4a. With HMGB1escalation, TLR2, TLR4, and their adaptor protein MYD88 were amplified significantly in RIR experienced animals, while anethole administration preceding to RIR significantly (*p* < 0.05) lowered mRNA expression of TLR2, TLR4, and their adaptor protein MYD88 (Figure 4b–d). When related to the sham group, nuclear NFκB p65 was significantly augmented in the RIR group, whereas NFκB p65 level was substantially diminished in rats received anethole compared to the RIR group in a dose-dependent manner (Figure 4e).

**Figure 5:**
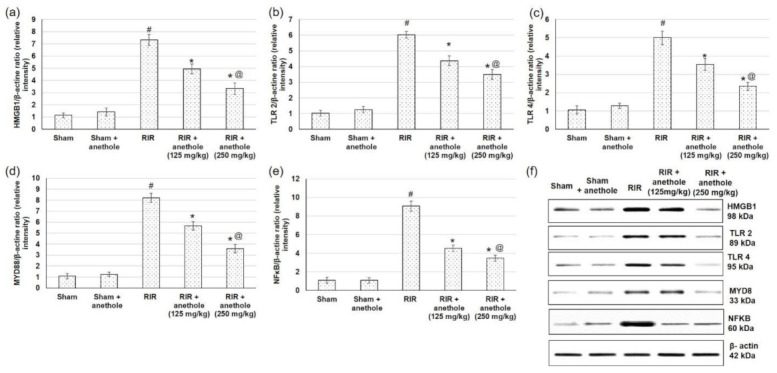
Effects of anethole (125 and 250 mg/kg) administration for 14 days prior to renal ischemia/perfusion (RIR) on the renal protein expression of (**a**) HMGB1, (**b**) TLR2, (**c**) TLR4, (**d**) MYD88, (**e**) NFκB and (**f**) protein expression of HMGB1,TLR2,4, MYD88 and NFκB in RIR-induced injury. All values are stated as mean ± SD. # designates statistically significant compared to sham group, * designates statistically significant compared to RIR group, and @ designates statistically significant compared to RIR + anethole 125 mg/kg group (*p* < 0.05) using one-way ANOVA followed by Tukey’s post hoc test.

As for protein expression (Figure 5), a similar pattern was obtained in which HMGB1 protein expression was augmented markedly (*p* < 0.05) in RIR related to sham, with subsequent augmentation in TLR2, TLR4, and MYD88. At the end of the studied pathway, NFκB showed higher levels in RIR related to sham (*p* < 0.05). On the other hand, anethole pretreatment prior to the renal ischemia/reperfusion lowered the HMGB1, subsequently TLR2, TLR4, and their adaptor protein MYD88, and finally NFκB (*p* < 0.05), signifying that the protective role of anethole could occur via the inhibition of the HMGB1/TLR2,4/MYD88/NFκB pathway. 

**Figure 6:**
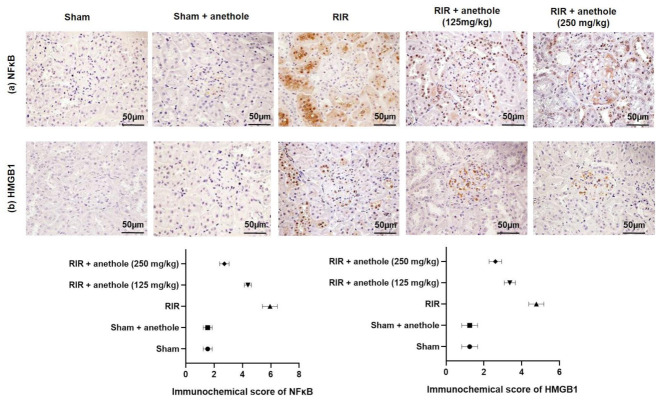
Effects of anethole (125 and 250 mg/kg) administration for 14 days prior to renal ischemia/perfusion (RIR) on the renal immunohistochemical assay of (**a**) NFκB and (**b**) HMGB1 in RIR-induced injury. All values are stated as mean ± SD.

As for the IHC analysis, the results revealed that NFκB (Figure 6a) and HMGB1 (Figure 6b) positive cells were infrequently present in the sham animals. Conversely, RIR renal tissues exhibited strongly HMGB1 and NFκB positive cell expression (*p* < 0.05). HMGB1 and NFκB positive cells were lowered in animal groups pretreated with anethole compared with the RIR group (*p* < 0.05). These results provide evidence that reno-protective effects of anethole could be via the inhibition of the HMGB1/TLR2, 4/MYD88/NFκB pathway.

### 3.5. In Silico Anethole Interaction with TLR

The in vivo outcomes of the current study suggests diminished expression levels for TLR2 and 4 and subsequent downstream genes (Figure 4) and proteins (Figure 5) as a mechanism to protect against RIR. However, another reason for the decreased levels of downstream mediators to TLRs is the direct effect of anethole on the TLRs. To explore this hypothesis, anethole was in silico docked against TLR 2 and 4. The results of the docking studies indicated weak interaction of anethole with TLR2 (data not shown); however, the compound showed deep interaction with TLR4. 

**Figure 7:**
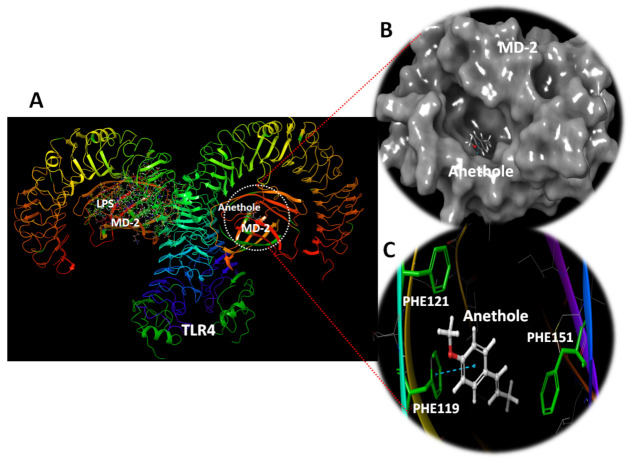
TLR4 complex with MD-2 exhibiting the docking site anethole. (**A**) The docking site of anethole in MD-2. The second MD-2 molecule was left bound with LPS for demonstration. (**B**) Surface depiction of MD-2 showing the docked anethole in the deep hydrophobic cavity of MD-2. (**C**) The docking site of anethole revealing its hydrophobic site comprising PHE119, PHE121, and PHE151. Stacking interaction is reported with PHE119.

In the TLR4, MD-2 forms a β-cup fold structure comprising antiparallel beta sheets, allowing a central deep hydrophobic cavity for ligand binding. The docking investigation with TLR4 demonstrates that anethole binds deep within the MD-2 hydrophobic pocket, indicating that it is a ligand for this receptor (Figure 7). Anethole was kept together by a hydrophobic cavity composed of the amino acids ILE46, VAL48, ILE52, LEU61, PHE119, PHE121, and PHE151. Hydrophobic interactions with PHE119, PHE121, and PHE151, as well as stacking interactions with PHE121, were discovered in the experiments.

### 3.6. Anethole Averts RIR Induced Renal Inflammation 

**Figure 8:**
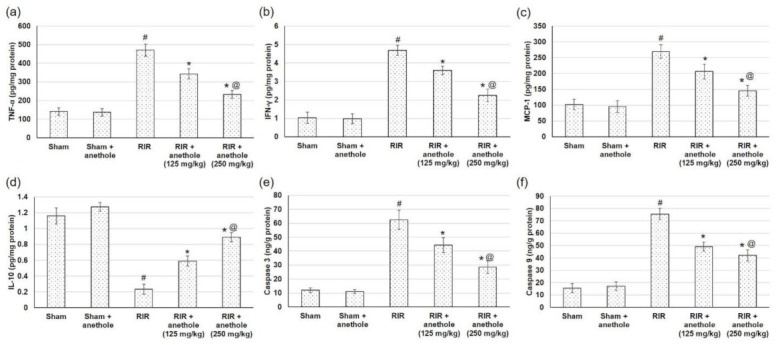
Effects of anethole (125 and 250 mg/kg) administration for 14 days prior to renal ischemia perfusion (RIR) on the renal inflammatory mediators (**a**) TNF-α, (**b**) IFN-γ, (**c**) MCP-1, and (**d**) IL-10, and on apoptosis, including (**e**) caspase 3 and (**f**) caspase 9, in RIR-induced injury. All values are stated as mean ± SD. # designates statistically significant compared to sham group, * designates statistically significant compared to RIR group, and @ designates statistically significant compared to RIR + anethole 125 mg/kg group (*p* < 0.05) using one-way ANOVA followed by Tukey’s post hoc test.

NFκB, an imperative downstream effector of TLR2/4 MyD88-dependent signaling, enhances proinflammatory responses. The current investigation inspected inflammation via the detection of several cytokines and inflammatory mediators. TNF-α, IFN-γ, and MCP-1 renal levels were amplified considerably (*p* < 0.05) in the RIR group, whereas anethole administration prior to renal ischemia/reperfusion distinctly prevented TNF-α, IFN-γ, and MCP-1 boost in RIR rats (Figure 8a–c, respectively). On the other hand, the level of IL-10, a cytokine that retains protective effects against inflammatory damage, was declined (*p* < 0.05) following RIR activity (Figure 8d). Conversely, anethole administration noticeably prohibited the RIR-induced reductions in IL-10. A higher concentration of anethole (250 mg/kg) exhibited reduced TNF-α, IFN-γ, and MCP-1 and augmented IL-10 levels compared to the lower dose of anethole (125 mg/kg). 

### 3.7. Anethole Averts RIR Induced Renal Function Apoptosis

Caspase 3 and 9, crucial members in apoptosis, were augmented subsequent to RIR injury (Figure 8e,f), while pretreatment with anethole lessened caspase 3 and 9 renal contents (*p* < 0.05) (Figure 8e,f), reflecting the anti-apoptotic actions of anethole, which may be related to the alleviation of RIR-induced injury. 

## 4. Discussion

Acute kidney injury (AKI), caused by renal ischemia/reperfusion RIR, is an imperative problem for nephrology consultants in hospitalized patients, during which a rapid decline in renal function evaluated via glomerular filtration rate (GFR) is observed [33]. In the current study, administration of anethole augmented Cr, BUN, uric acid, LDH, and Kim-1 levels in RIR animals, indicating progressive renal damage. These results are in accordance with previous studies demonstrating progressive renal injury associated with RIR [2,5,7,33]. Reoxygenation of the ischemic tissue triggers ROS generation, which exceed the level of the endogenous antioxidant enzymes [2,3]. Indeed, the outcomes from the current study showed depletion of antioxidant enzymes including SOD, CAT, and GPx, and GSH content in the RIR group. Additionally, RIR-experienced animals exhibited lipid peroxidation amplification, as evidence by higher MDA levels. This could be due to the capability of ROS to react with proteins, lipids, and nucleic acids, leading to lipid peroxidation of the cell membranes. Several studies have demonstrated that RIR is associated with lipid peroxidation, causing oxidative damage of the cellular membranes [2,5,7].

With HMGB1 intensification, TLR2, TLR4, and their adaptor protein MYD88 and subsequently NFκB gene and protein expression levels were all amplified in RIR experienced animals. HMGB1 is crucial in mediating apoptosis as well as inflammation in renal injury [33]. Studies demonstrated that HMGB1 plays an important role in the interaction of autophagy and apoptosis/necrosis in various disorders, including renal ischemia/reperfusion [7]. Wu et al. (2010) [9] verified that mice treated with anti-HMGB1 antibody exhibited protection against RIR injury development. Additionally, anti-HMGB1-treated RIR animals demonstrated low inflammatory mediators levels. On the contrary, HMGB1 agonist (rHMGB1) administration after renal ischemia/reperfusion aggravated RIR injury in wild-type mice [9].

HMGB1 triggers cellular signaling through TLR, especially TLR2 and TLR4, leading to proinflammatory cytokine and chemokine augmentation, causing RIR damage [9]. TLR4 expressed within the kidney is a potential mediator of innate activation and inflammation. Wu et al. (2007) [8] confirmed TLR4 expression intensification by tubular epithelial cells and infiltrating leukocytes subsequent to renal ischemia. MyD88 interacts with the IRAK complex, prompting further signaling pathways, including NFκB instigation [34]. Moreover, TLR4^–/–^ and MyD88^–/–^ mice exhibited protection against renal dysfunction following RIR [8]. Another study verified that genetic elimination of both TLR2 and TLR4 offered a comparable defense following RIR injury when related to single removal of TLR2 and TLR4 [12]. During RIR-induced injury, TLR2 and TLR4 were amplified to activate the intracellular signaling cascade through NFκB. Subsequently IL-1β, IL-6, IL-18, MCP1, IFN-γ, and TNF-α are intensified, leading to acute tubulointerstitial injury [11,12,28,34].

In the existing study, the RIR group showed elevated levels of HMGB1, which in turn activated TLR 2, TLR 4, and NFκB, and subsequently inflammatory factors (MCP1, IFN-γ and TNF-α) were activated. Numerous stimuli, including ischemia/reperfusion injury, activate NFκB signaling by the degradation of IκB and release of the NFκB p65-p50 dimer, which translocates to the nucleus, binds to κB binding sites on DNA, and regulates the transcriptional activation of target genes [7]. Furthermore, RIR injury augmented caspase 3 and 9, indicating apoptotic status arising within renal tissue. Previous studies demonstrated an extensive intensification in renal apoptosis following RIR [5,9,33,34].

Medicinal plants have been the basis of conventional medicine for hundreds of years and often a foremost foundation of pharmacological active constituents. Plant essential oils and their constituents are being utilized in both foods and pharmaceuticals. Anethole is extensively used in cosmetic, perfumery, and pharmaceutical industries and is employed as a flavoring additive in food manufacturing. Various studies have disclosed several pharmacological actions of anethole, for instance anti-inflammatory [15,17], anticarcinogenic [16], chemopreventive, hepatoprotective [22], antidiabetic [35], neuroprotective [23], and immune-modulatory [14]. All these pharmacological actions are due to the modulation of several cell signaling pathways. As for renal action of anethole, recently Samadi-Noshahr et al. (2021) [36] showed that trans-anethole diminished kidney injury as well as lowered angiotensin II receptor (AT1R) and TGF-β expressions in streptozotocin (STZ)-induced diabetic rats. However, the actions of anethole on RIR have never been studied. 

Management with anethole prior to RIR showed enhanced renal morphology and improved renal function, as evidenced by the lowered levels of Cr, BUN, uric acid, LDL, and Kim-1 when related to the RIR group. These results postulate that anethole averts RIR-induced renal function alterations and histological changes. Previously, trans-anethole showed the protective effects on the GFR, protein, urine production, and kidney/body weight ratio, as well as lowered tubule vascular degeneration, and glomerular and tubulointerstitial sclerosis in STZ diabetic animals [36]. 

As for the antioxidant effects, anethole amplified GSH renal content and SOD, CAT, and GPx activities and prohibited the amplified levels of MDA, indicating lower lipid peroxidation. These results together suggest that RIR-induced oxidative stress might be managed by anethole due to its efficient antioxidant ability. Anethole inhibited TNF-α induced by both lipid peroxidation and ROS generation [37]. Furthermore, pretreatment with anethole lowered the expression levels of HMGB1, TLR2, TLR4, and MYD88 and finally NFκB. These results are evidence that the reno-protective effects of anethole could occur via the inhibition of the HMGB1/TLR2, 4/MYD88/NFκB pathway. Previously, Cho et al. (2013) [22] showed the protective mechanism of trans-anethole against liver ischemia/reperfusion damage in mice via inhibition of the release of HMGB1 into the extracellular milieu, and the interactions between interferon regulatory factor (IRF)-1 and histone acetyl transferase. Similarly, trans-anethole suppressed the elevated TLR4, MYD88 protein expression, phosphorylation of p38, and nuclear translocation of NFκB. Anethole administration prior to RIR distinctly prevented TNF-α, IFN-γ, and MCP-1 boost in RIR rats, whereas it prohibited the RIR-induced reductions in IL-10. For the anti-inflammatory actions of anethole, Ritter et al. (2013) [15] proved that anethole lowered neutrophil recruitment in carrageenan and complete Freund’s adjuvant (CFA)-induced inflammation. Anethole deterred TNF-α and IL-1β in response to carrageenan and TNF-α, IL-1β, and IL-17 in response to CFA [15]. Chainy et al. (2000) [37] verified that anethole exhibited a potent inhibitory action on TNF-induced NFκB activation. Anethole reduced matrix metalloproteinase (MMP)-2 and -9 expression and suppressed the phosphorylation of AKT, extracellular signal-regulated kinase (ERK), p38, and nuclear transcription of NFκB in HT-1080 cells, suggesting that the compound inhibited ERK/p38 MAPK/AKT/NFκB signaling [16]. In the current study, management with anethole prior to RIR lessened caspase 3 and 9, reflecting the anti-apoptotic actions of anethole, which may be related to the alleviation of RIR-induced injury. An earlier report showed that anethole abrogated TNF-induced apoptosis as measured by both caspase activation and cell viability [37]. 

Computational studies suggested the binding of anethole with the deep hydrophobic pocket of MD-2, which might interfere with LPS and binding of other MD-2 ligands. This suggests that anethole might modulate MD-2-TLR4 interactions and affect downstream signaling, allowing for an additive anti-inflammatory response.

## 5. Conclusions

Renal ischemia/reperfusion (RIR)-experienced animals exhibited progressive renal injury as evidenced by lowered renal function and alterations in histology examination. RIR induced severe oxidative, inflammatory, and apoptotic status within renal tissue. Management with anethole prior to RIR showed enhanced renal morphology and improved renal function. Anethole amplified GSH renal content and SOD, CAT, and GPx activities, and lowered MDA levels, indicating inferior lipid peroxidation and superior antioxidant enzyme activities. The intake of anethole prior to RIR diminished the expression levels of HMGB1, TLR2, TLR4, MYD88, and NFκB, indicating that the nephro-protective effect of anethole could occur via the inhibition of the HMGB1/TLR2, 4/MYD88/NFκB pathway. Pretreatment with anethole administration declined TNF-α, IFN-γ, MCP-1, and caspase 3 and 9, whereas it increased IL-10, reflecting the anti-inflammatory and anti-apoptotic actions of anethole. These outcomes could support the use of anethole, or the compound-containing plant essential oils, to protect against RIR in susceptible patients.

## Figures and Tables

**Table 1:** Animal experimental design. The differences between the five groups of animals specified in the experimental design section.

Animal Experimental Groups	1% CMC in Saline Orally (for 14 Days before Surgery)	Anethole Orally # (for 14 Days before Surgery)		Renal Arteries Clamping (for 45 Min, then Reperfusion)
		125 mg/kg	250 mg/kg	
Sham	Yes	No	No	No
Sham + anethole	No	No	Yes	No
RIR	Yes	No	No	Yes
RIR + anethole (125 mg/kg)	No	Yes	No	Yes
RIR + anethole (250 mg/kg)	No	No	Yes	Yes

# Anethole was dissolved in 1% carboxymethylcellulose (CMC) in saline.

**Table 2:** Primer sequences used for qPCR of TLR pathway gene expression.

TLR Pathway	Primer Sequence (5′ to 3′)	Gen-Bank Accession Number
HMGB-1	5′-AGGCTGACAAGGCTCGTTATG-3′ (sense) 5′-TGTCATCCGCAGCAGTGTTG-3′ (antisense)	XM_039100270
TLR2	5′-ATGAACACTAAGACATACCTGGAG-3′ (sense) 5′-CAAGACAGAAACAGGGTGGAG-3′ (antisense)	NM_198769
TLR4	5′-CATGACATCCCTTATTCAACCAAG-3′ (sense), 5′-GCCATGCCTTGTCTTCAATTG-3′ (antisense)	NM_019178
MyD88	5′-GAGATCCGCGAGTTTGAGAC-3′ (sense) 5′-CTGTTTCTGCTGGTTGCGTA-3′ (antisense)	NM_198130.2
NFκB	5′-ATCATCAACATGAGAAACGATCTGTA-3′ (sense) 5′-CAGCGGTCCAGAAGACTCAG-3′ (antisense)	L26267.1
β-Actin	5′-TGCTATGTT GCCCTAGACTTCG-3′ (sense) 5′-GTTGGCATAGAG GTCTTTACGG-3′ (antisense)	NM_031144

## Data Availability

The authors confirm that the data supporting the findings of this study are available upon request.
